# From sea monsters to charismatic megafauna: Changes in perception and use of large marine animals

**DOI:** 10.1371/journal.pone.0226810

**Published:** 2019-12-31

**Authors:** Carlotta Mazzoldi, Giovanni Bearzi, Cristina Brito, Inês Carvalho, Elena Desiderà, Lara Endrizzi, Luis Freitas, Eva Giacomello, Ioannis Giovos, Paolo Guidetti, Adriana Ressurreição, Malcolm Tull, Alison MacDiarmid

**Affiliations:** 1 Department of Biology, University of Padova, Padova, Italy; 2 CoNISMa (Interuniversitary Consortium of Marine Sciences), Rome, Italy; 3 Dolphin Biology and Conservation, Cordenons, Italy; 4 CHAM—Center for the Humanities, NOVA FCSH/Uaç, Lisbon, Portugal; 5 Associação para as Ciências do Mar, APCM, Lisbon, Portugal; 6 Instituto Gulbenkian de Ciência, IGC, Oeiras, Portugal; 7 Université Côte d’Azur, CNRS, UMR 7035 ECOSEAS, Nice, France; 8 Museu da Baleia da Madeira, Caniçal, Madeira, Portugal; 9 MARE–Marine and Environmental Sciences Centre, Horta, Portugal; 10 IMAR-Instituto do Mar, Horta, Portugal; 11 OKEANOS Centre, University of the Azores, Horta, Portugal; 12 iSea, Environmental Organisation for the Preservation of the Aquatic Ecosystems, Greece; 13 CCMAR Centre of Marine Sciences, Faro, Portugal; 14 Murdoch University, Perth, Australia; 15 NIWA, Wellington, New Zealand; Universita degli Studi di Genova, ITALY

## Abstract

Marine megafauna has always elicited contrasting feelings. In the past, large marine animals were often depicted as fantastic mythological creatures and dangerous monsters, while also arousing human curiosity. Marine megafauna has been a valuable resource to exploit, leading to the collapse of populations and local extinctions. In addition, some species have been perceived as competitors of fishers for marine resources and were often actively culled. Since the 1970s, there has been a change in the perception and use of megafauna. The growth of marine tourism, increasingly oriented towards the observation of wildlife, has driven a shift from extractive to non-extractive use, supporting the conservation of at least some species of marine megafauna. In this paper, we review and compare the changes in the perception and use of three megafaunal groups, cetaceans, elasmobranchs and groupers, with a special focus on European cultures. We highlight the main drivers and the timing of these changes, compare different taxonomic groups and species, and highlight the implications for management and conservation. One of the main drivers of the shift in perception, shared by all the three groups of megafauna, has been a general increase in curiosity towards wildlife, stimulated *inter alia* by documentaries (from the early 1970s onwards), and also promoted by easy access to scuba diving. At the same time, environmental campaigns have been developed to raise public awareness regarding marine wildlife, especially cetaceans, a process greatly facilitated by the rise of Internet and the World Wide Web. Currently, all the three groups (cetaceans, elasmobranchs and groupers) may represent valuable resources for ecotourism. Strikingly, the economic value of live specimens may exceed their value for human consumption. A further change in perception involving all the three groups is related to a growing understanding and appreciation of their key ecological role. The shift from extractive to non-extractive use has the potential for promoting species conservation and local economic growth. However, the change in use may not benefit the original stakeholders (e.g. fishers or whalers) and there may therefore be a case for providing compensation for disadvantaged stakeholders. Moreover, it is increasingly clear that even non-extractive use may have a negative impact on marine megafauna, therefore regulations are needed.

## Introduction

The conservation of marine megafauna is crucial to preserve healthy and balanced ecosystems [1–5]. In the past, the westernized world regarded various species as creatures shrouded in mystery, dangerous monsters, or resources for the exclusive benefit of humankind [6–10]. In Europe, for instance, hunting, gathering and fishing have been important means of livelihood and survival in coastal societies [11]. Although the exploitation of sea resources by native peoples was more sustainable in the past, nonetheless examples of the extinction, extirpation or severe decline of marine megafauna are to be found from the 11th century on [12]. Nonetheless, only in recent decades has the human overexploitation of marine megafauna emerged as a significant issue [12, 13].

Today, most megafauna species are considered as charismatic animals and flagships species, i.e., species that have “the ability to capture the imagination of the public and induce people to support conservation action and/or to donate funds” [14]. These species have the potential for raising public and institutional awareness, promoting conservation actions that may benefit other species and the entire marine ecosystem. Human perception of charismatic species has changed considerably over time. This change has been influenced *inter alia* by organizations for environmental protection, science education and, in particular, ocean literacy, aquaria, wildlife documentaries, books, movies and cartoons, and the actions of committed individual scientists as well as enlightened policy makers and spokespersons [15–17].

As is generally true for wildlife, the change in the perception of marine flagship species has been paralleled by a change in use, from extractive (i.e. fishing or hunting) to non-extractive (i.e. watching), with an important role played by ecotourism. Since the 1990s, ecotourism has been a growing industry involving increasing numbers of participants and stakeholders, and of growing economic value [18, 19]. The increasing importance of ecotourism, and its pitfalls and benefits for the environment, have been recognized by the United Nations, which designated 2002 ‘International Year of Ecotourism’ [20], and, more recently, 2017 as the 'International Year of Sustainable Tourism for Development' (resolution 70/193, 22^nd^ December 2015; United Nations). Ecotourism was defined by Hetzer in 1965 as a type of tourism that minimizes environmental impacts and the impact on host cultures, provides maximum economic benefits to local communities and maximum recreational satisfaction to participating tourists [21]. More recently, in 2015, the International Ecotourism Society defined it as "responsible travel to natural areas that conserves the environment and improves the well-being of local people". Although there are several other definitions of ecotourism, this industry is generally characterized by a 'nature-based' approach. Interest in wildlife, pristine landscapes, and direct contact with nature have led to the development of activities such as wildlife watching, nature photography, diving and trekking [18]. While mass ecotourism has its downside and may negatively impact wildlife and the environment [19, 22], the industry represents a powerful driver to push environmental conservation, and to raise public awareness [18].

In marine environments, wildlife-focused tourism is linked with the growth of marine recreational activities including scuba diving, snorkeling and watching marine wildlife from boats and from land. In addition, an increasing number of 'citizen science' projects are dedicated to the monitoring of marine life, particularly protected species [23]. The watching of marine wildlife is primarily focused on charismatic species such as marine mammals (particularly cetaceans), and more recently elasmobranch species [23, 24].

The change in the perception and use of some marine animals in certain geographical areas supports tourism industries that, eventually, have generated more economic wealth than extractive industries [25, 26]. Some cetacean and sea turtle species that are currently the target of ecotourism were previously close to extinction, and the change of perception and the economic potential of ecotourism has contributed to their protection or recovery [25].

Here, we describe the changes in the human perception, use and value of the three main groups of marine megafauna: cetaceans, elasmobranchs and groupers. We highlight the drivers of these changes in order to better understand why the societal value has changed for some species and not for others. For that purpose, we have used the published literature on the topic and also documental sources (written and iconographic) in a search for evidence of changes in the use of marine species; we have focused mostly on European waters, cultures and history, while also providing some extra-European examples for comparison.

Specifically, our aims were to: 1) evaluate the changes in the perception and use—from extractive to non-extractive—of marine megafauna based on case studies of cetaceans, elasmobranchs and grouper; 2) highlight the main drivers and the timing of this change; 3) compare different taxonomic groups and species in the light of the drivers; and 4) highlight the related management and conservation issues.

### Cetaceans

#### Historical perspectives and human perceptions

Human perception of cetaceans—the infraorder that includes whales, dolphins and porpoises—is very diverse according to local practices, cultures and worldviews. In westernized societies, it has changed dramatically over the centuries, with important differences according to geographical areas and species (particularly in relation to size, economic value, ease of capture and degree of conflict with fisheries).

Since time immemorial, whales have fascinated humans, prompting the growth of a whale mythology inspired by the mystery surrounding these creatures. 'Sea monster' iconography was common in Greek, Etruscan, and Roman representations [10]. In medieval and early modern European textual traditions, whales were perceived as aggressive and dangerous creatures to fear and avoid, but at the same time as valuable resources [6, 7]. Most perceptions have been consistently negative and predatory, promoting fear and the dominance of humans over whales [7].

**Fig 1:**
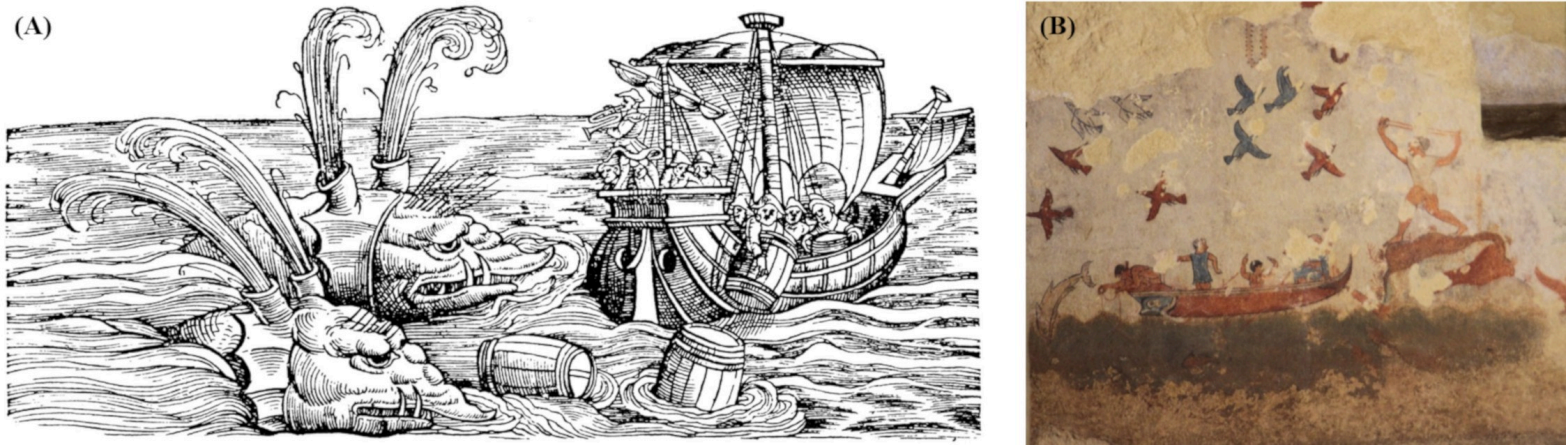
(A) Representation from the 16th century of two whales attacking a ship, source [27]; (B) Etruscan painting from the tomb of hunting and fishery (Necropolis of Tarquinia, Italy), reproduced under a CC BY license, by permission of the photographer Tea Giomi.

Until the 15th century, whales were depicted as monstrous beasts, and the subject of a rich and highly imaginative iconography (Fig 1A; though some representations were relatively more realistic).

**Fig 2:**
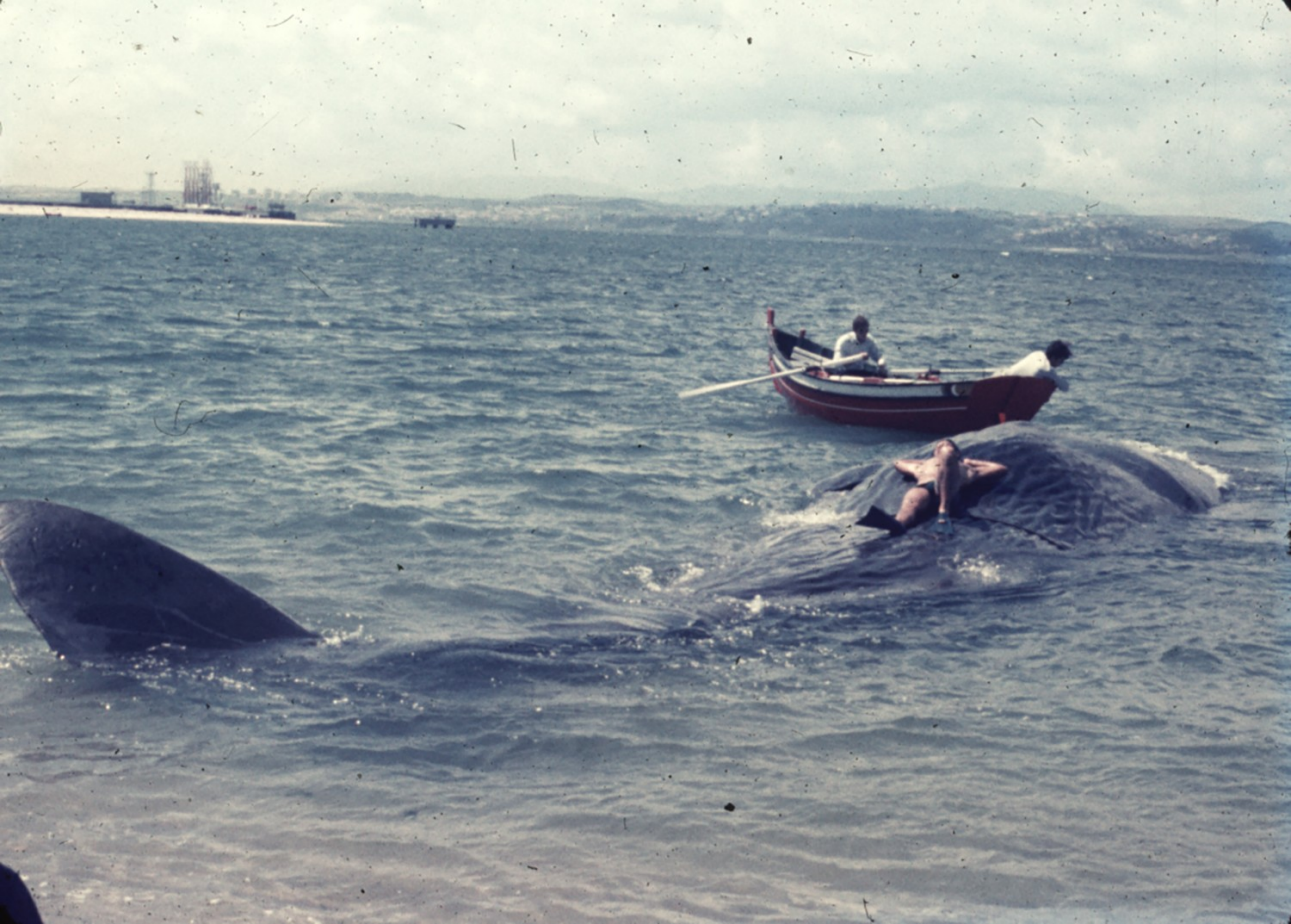
Local people's interactions with a sperm whale stranded in the Tagus estuary (Lisbon, Portugal). Mid-20th century photograph, reproduced under a CC BY license, by permission of the photographer, Carlos Carvalho.

Furthermore, until at least the 17th century, cetacean strandings could be viewed as divine messages and given moralistic interpretations, e.g. considered as a bad omen and linked to disasters and tragedies such as wars, shipwrecks and earthquakes. For centuries, responses to cetacean live strandings—typically including the killing or harming of the animals—were either utilitarian or characterized by feelings including fear and a desire to "subjugate the beast", with no apparent concern for the animal's suffering and death [6]. The perception of the stranded beast’s economic value emerged very early on European shores; scavenging of both dead and live stranded whales was a common practice [28]. While in the Mediterranean Sea there was never an extensive commercial whaling tradition [29], since medieval times most whales that were stranded alive or approached the coast were promptly butchered and used to extract oil, and sometimes also used for scientific purposes or display [30]. In the first half of the 20th century, behavior towards large cetaceans stranded alive was still characterized by open hostility. In the Adriatic Sea, for instance, almost all cases of live stranding of whales with a known human response involved attempts at killing [6, 30, 31]. Sperm whales (*Physeter macrocephalus*) and at least one fin whale (*Balaenoptera physalus*) stranded alive in the Adriatic Sea were harpooned, shot, machine-gunned, injured by explosives, roped, and sometimes used to extract oil [30, 31]. In different areas, including the Adriatic Sea and the shores of the Western Atlantic (Fig 2), it was even customary to pose for group photos on top of stranded dead whales, often smiling or standing in a fierce pose.

On the other hand, strandings attracted much interest across medieval, early modern and modern Europe, as expressed in literature, poetry, paintings, daily news, pamphlets, displays, natural history collections and museums [32], and cetaceans became increasingly more widely acknowledged and known.

Representations of cetaceans became more accurate over time, whales progressively shifting (? progressing) from the status of monsters and freaks to that of animals that are worthy of scientific interest [6, 7, 32, 33].

Herman Melville’s 1851 novel *Moby Dick* about the relationship between a whaling captain and a sperm whale marks this transition, highlighting both the freakish nature of the white whale (a sperm whale), and the need to understand and appreciate its role in the marine environment. However, it was only in the 1970s and 1980s that attitudes towards whales changed drastically due to a combination of increased awareness, a decline in the use of cetaceans as a resource, and the emergence of some degree of biophilia [34]. Documentary films played a pivotal role in this shift [35, 36]. This change in perception also involved the attitude towards strandings. A major shift in perception appears to have started in the 1970s; by the 1980s, killings in the Mediterranean and elsewhere were consistently replaced by efforts to rescue the animals. In 1984, a sperm whale stranded near Pescara, Italy, was the subject of considerable attention among the public and the media. The animal was described as a "amiable" and "intelligent" creature in local newspapers, with criticism of the failure of the rescue attempt and sadness over the death of a "king of the seas" [6].

Dolphins were generally perceived differently from whales. For instance, Greek and Etruscan cultures often represented dolphins in paintings, mosaics and on jars, jumping and swimming close to boats and positively interacting with people (Fig 1B). In Greek mythology, a god, Apollo, assumed the form of a dolphin; Poseidon was often represented with a dolphin. Friendly human-dolphin contacts are reported in Roman legends, and on an Australian island, Aborigines believed that direct communication between dolphins and a medicine man would give to the tribe fortune and happiness [37]. While not being generally perceived as animals dangerous to humans, dolphins were nonetheless not immune from being hunted or actively culled.

In the Mediterranean Sea, for instance, dolphins have long been viewed not only as a food resource or target of recreational fishing [38], but also as pests deserving systematic extermination, due to their role of fish predators. Deliberate dolphin killings since historical times were largely attempts to reduce perceived conflict with fisheries. In the 19th century, killing the largest possible number of dolphins was a major concern of fishery managers [39]. Dolphins were depicted as "ichthyophagous monsters", "phony and noxious pirates" and "man's worst enemies" [40]. Culling campaigns were a widespread activity in the past, with at least 14 countries supporting such programs [41], including Norway, Spain, France, Italy, Greece, and the former Yugoslavia. Offering bounty was a common way of promoting dolphin killings [41–45]. Culling has been particularly well documented in the Adriatic Sea, where common dolphins and common bottlenose dolphins were killed by the thousand [43] and in the Black Sea, where about 6 million dolphins and porpoises were culled in the 20^th^ century [46]. Monetary rewards were offered for each animal killed and landed, on both the eastern and the western sides of the Adriatic, as well as in Greece. Killing dolphins for bounty, human consumption or sport remained a common practice until the 1960s, and it was only in 1979 that the Italian Government prohibited unauthorized dolphin killings, whereas in Greece killings remained legal until 1980 [44] and in Croatia until 1995 [42, 43].

Killing small cetaceans was also common practice among scientists and cetacean research pioneers until the 1960s. In the 1960s and 1970s, dolphins were also routinely caught in the Mediterranean and the Black Sea for live display in captive facilities [46–48], as still happens today in several parts of the world.

For both baleen whales and toothed cetaceans, increasingly accurate information concerning their biology and behavior has become available since the 1970s, which has certainly contributed to changing public perceptions and reducing hostility and fear [49–51]. Scientific knowledge informed this process through the discovery of whale songs [52], cetacean cognitive abilities [53] and several other aspects of cetacean behavior [54]. Pseudo-science (i.e. statements that do not adhere to accepted scientific standards) inferences of a highly developed intelligence and supernatural abilities [55, 56] may have also influenced public perceptions. TV documentaries, television series and movies in the 1970s (e.g. the NBC series *Flipper'* between 1964 and 1967, or Mike Nichol's science-fiction thriller *The Day of the Dolphin* in 1973) started portraying dolphins as peaceful and harmless, and popular books and magazines featured photos and articles celebrating whales and dolphins in their natural environment. Animal rights movements and environmental organizations also contributed to increased awareness and appreciation of cetaceans, also leading to the establishment of marine protected areas and sanctuaries specifically created for cetaceans (e.g. the Pelagos Sanctuary in the north-western Mediterranean Sea). The general empathy of the public-at-large with these animals helped in the development of conservation programs, by increasing awareness and commitment [50, 51]. This also led to the increasing development of industries related to the observation of wild populations of cetaceans that, if not properly regulated, can also severely impact the animals.

#### Shift from extractive to non-extractive industries

For centuries, whales have been hunted for their valuable oil, meat, bones and baleen, with whale killing methods and processing techniques developing over time [7, 57]. Here, we focus on the changes in the uses of whales over time in Europe: from biblical Leviathan to valuable natural resources [7, 58], conservation icons, and tourism attractions [59]. Perhaps the first westerners to hunt large whales in an organized and deliberate manner were the Basques, who set industrial standards that lasted for hundreds of years [60, 61]. Whaling used to be an important economic activity for many countries around the globe, due to the extremely high profitability of the whale oil market [62]. One of the main targets, the North Atlantic right whale *Eubalaena glacialis*, was decimated in the eastern North Atlantic by the 17^th^ or 18^th^ century [63].

In Iberia, whaling peaked in the industrial age during the late 19^th^ and early to mid-20^th^ centuries. It was in the same period that Australia, Tasmania, and New Zealand also embraced shore-based and pelagic whaling activities together with certain regions in the USA, Norway and Iceland [64]. During the 20^th^ century in particular, shore-based industrial whaling took place in northern and southwestern Spain (e.g. [65, 66]) as well as in Portugal [67, 68], Brazil, Cape Verde, USA, Australia and Norway ([64] and all the references therein). In Portugal (including its Atlantic archipelagic waters), almost 30,000 whales were landed over 91 years of whaling, from 1896 to 1987 [69]. In Australia and New Zealand, the total catches of southern right whales from the 19^th^ century onwards was estimated to be between 53,000 and 58,000 [70].

In addition to the culling of dolphins, as a result of the perceived competition with fishermen, dolphins too have been exploited in several countries since prehistoric times (e.g. [71] for their meat and also for the trade of some parts such as teeth (e.g. [72]. However, the extractive industry for dolphins was not as widespread and organized as that of whales, and global or even local data are scanty. Nonetheless, dolphins are still today part of the aquatic bushmeat in various African and South American countries [73].

**Fig 3:**
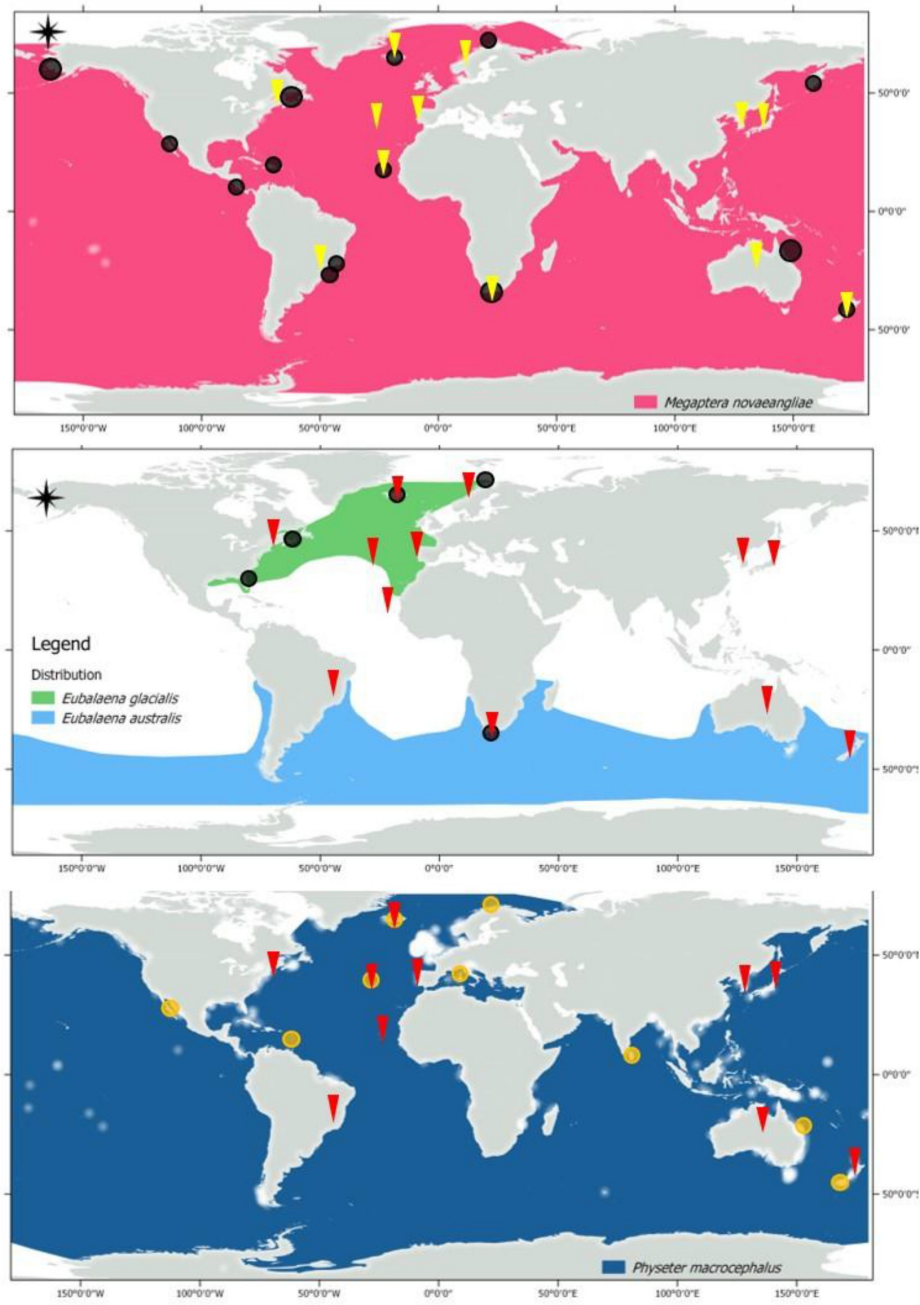
Worldwide distribution of sperm whale (*Physeter macrocephalus*), North Atlantic right whale (*Eubalaena glacialis*), southern right whale (*Eubalaena australis*) and humpback whale (*Megaptera novaeangliae*), based on the shapefiles obtained from the IUCN website (http://www.iucnredlist.org/technical-documents/red-list-training/iucnspatialresources). The circles represent current whale-watching hotspots obtained through [59], complemented with an online search. Most of these circled areas overlap with former shore-based whaling grounds.

The shift towards a non-extractive whale- and dolphin-based industry dates back to 1955 in California, when whale watching started [74]. The term of whale watching has been used to represent cetacean watching in general, thus including whales, dolphins and porpoises in the wild [75]. Initially, until the late 1970s, this activity was performed mainly on shore, while later whale and dolphin watching from boats became more and more popular [74]. During the 1980s, the whale-watching industry was mainly limited to a few countries, but then it spread worldwide (Fig 3), involving at least 58 cetacean species (S1 Table), increasing exponentially in terms of numbers of tourists involved, creating significant economic and social opportunities [74–78], as an alternative source of livelihood for whaling communities.

The cultural heritage of whaling traditions promoted the assembly of whale collections and the creation of dedicated museums (e.g. the whale and whaling museums in Madeira and the Azores, the Husavik Whale Museum in Iceland, or the New Bedford Whaling Museum in the US), aimed at preserving the history and heritage of whaling, while also promoting scientific research. Whaling museums also support educational programs and raise conservation awareness [79, 80].

#### Implications for conservation and management

Over time, and across regions, whale hunting drove the decline of whale populations and some species reached the brink of extinction. By the 1930s, many whale populations were brought to near collapse by intensive whaling [81, 82]. This triggered the creation of the International Whaling Commission (IWC) in 1946, to enable “the proper conservation of whale stocks and thus make possible the orderly development of the whaling industry” [83]. However, in the first decade of its existence, the IWC failed to fulfill its objectives, and many whale species were still facing extinction by the 1980s. The prospect of imminent extinctions, along with a growing anti-whaling movement and the inclusion of several anti-whaling states as members of the commission, led the IWC in 1982 to adopt a moratorium to pause commercial whaling (except for aboriginal communities and scientific research). Since then, there has been a significant global reduction in whale kills and whale meat consumption, with an improvement in the conservation status of at least some populations [84]. For instance, whaling has been banned in mainland Portugal, Azores and Madeira (in 1981, 1984 and 1986, respectively). The last sperm whales were illegally captured in the Azores in 1987. In Madeira, cetacean ecotourism started in 1995 and since then it has become an important industry. Similar transitions from the abandonment of declining commercial whaling to the protection of marine mammals, and the emergence of a thriving whale-watching business, have occurred in the USA, South Africa, Australia and New Zealand. This industry is also emerging in countries such as China, Cambodia, Laos, Nicaragua or Panama [75]. However, in countries such as Norway, Iceland and Japan, whaling still co-exists with whale watching [85].

Similarly, the capture of small cetaceans was banned through international and national legislation, even though dolphins may still be captured as bushmeat in some parts of the Atlantic ([73] and all the references within) as well as in other regions [86]. Moreover, dolphins may be caught as by-catch in fisheries targeting other species. In the 1970s there was, for example, great concern about the high by-catch of dolphins by purse seines deployed to catch tuna. The issue arose firstly in the USA, leading to the development of campaigns, promoted by environmental organizations, aimed at raising public awareness and pushing towards the adoption of fishing methods able to produce 'dolphin-free' tuna [87]. Among the actions undertaken, in the 1970s the so-called Medina panels were introduced in purse seines to prevent dolphins from becoming entangled [88].

Following the global IWC ban on commercial whaling, the declining whale populations started to slowly recover, occupying former areas of distribution and eventually turning into flagship species. This recovery in abundance of whales (and other cetaceans) can lead to changes in the ecosystems and to the recovery of habitats and the restoration of ecosystem services [89]. Sperm whales *Physeter macrocephalus* and humpback whales *Megaptera novaeangliae* exert strong top down controls on pelagic and coastal prey species [90, 91]. In addition, cetaceans can enhance primary productivity in feeding areas by concentrating nitrogen near the surface through the release of fecal plumes. This upward ‘'whale pump'‘ played a much greater role before the whaling era, when marine mammal recycling of nitrogen could be more than three times the atmospheric nitrogen input. Even with today's much reduced populations, marine mammals provide an important ecosystem service by sustaining productivity in regions where they occur in sufficiently high densities [92]. As some whale populations approach pre-harvesting levels, we can expect to see a rise in associated ecosystem services along with conflict, real and perceived, with human activities such as commercial fisheries [89]. Awareness-raising efforts should continue primarily targeting the fishing community, as dolphins are still considered as competitors in many areas, even though they have been protected for more than two decades. At the same time, whale and dolphin watching have emerged as a (more) sustainable way of once again using these animals as a valuable economic resource and bringing them closer to people’s lives. In fact, at present, the most significant non-extractive use involves whale- and dolphin-watching in its multiple formats (from land, from boats, swimming with the animals). This has become an important business activity for some communities, bringing in considerable sums in revenue and significant numbers of jobs [93].

The shift from an extractive to a non-extractive industry for cetaceans is not exempt from potential negative effects on the welfare of wild animals. The watching of wildlife has often been referred as a 'non-consumptive' use, i.e. a use of a resource in ways that do not reduce its supply. Some authors have questioned the use of the term 'non-consumptive' in the context of whale watching (see [94] for a discussion on this issue) since there is evidence that the intensification of whale-watching activities may have a negative impact on cetaceans, altering their short and/or long-term behavior patterns and leading to displacement, progressively affecting the animals’ health and therefore their survival [95–98]. Current conservation measures should therefore include not only population management, but also the management/regulation/control of human activities that can affect whales and dolphins. Although the impact of whale-watching represents a significant threat for cetaceans, other activities have a more severe impact on their populations. Habitat degradation [43], intentional killing as retaliation from fishers [86], incidental fishing [99], noise pollution [100] and the use of some species as bait [101] are among the main threats to cetaceans nowadays. Despite the intensive research efforts made during the last decades, knowledge regarding the status of some species in general and/or in particular areas is still lacking ([102] and references therein).

### Elasmobranchs

#### Historical perspectives and human perceptions

Elasmobranchs, and in particular sharks, have always been perceived as dangerous animals. Since ancient times, iconography and literature represented them as ferocious animals, man-eaters and voracious predators [9, 103, 104]. Even more recently, movies and cartoons offer a frightening representation of these species. It is worth recalling the well-known book *Jaws*, written by Peter Benchley in 1974, telling the story of attacks by a great white shark. The book was a great success and based on it, a movie with the same title was released in 1975 ($260,000,000 total revenue). Since then, several movies with sharks as the main character have been produced, e.g. *Shark*, *Jaws 2*, *Deep Blue Sea*, *The Shallows* and *The Meg*, with the vast majority presenting sharks as monstrous and vengeful creatures. Even in old children’s literature, sharks were commonly represented as savage beasts, e.g. the “terrible dogfish” in the novel *The Adventures of Pinocchio* by Carlo Collodi in 1883. Nevertheless, a few recent movies and children’s books display a remarkable reverse, depicting sharks as naïve and sympathetic animals, especially when they refuse to keep eating fish (e.g. *Shark Tale*, *Nemo*, *Surprising Sharks*, *Zig & Sharko*).

The fear for sharks is closely related to stories of shark attacks, the occurrence of which worldwide has always received great attention from the media. After the Second World War, the increase in bathing in marine areas led to more potential contact between people and sharks [104]. At the same time, the increase in the use of mass media enabled the widespread distribution of information about shark attacks and the potential to influence public opinion regarding these animals. The mass media, and even some scientific opinion, tended to ‘criminalize’ shark attacks, depicting 'brutal' sharks attacking humans [102, 103]. Even if in more recent times, scientists have been trying to change the popular perception of shark attacks, promoting a more objective reclassification of human–shark interactions, nonetheless the resonance of attacks as reported by the media enhances feelings of fear towards these species [105]. This attitude may promote shark-culling policies [104, 106, 107], and vigorous debate between environmentalists and people afraid of sharks. An interesting case study is that of Australia. As more and more Australians engaged in water-related activities, shark attacks have increased, especially since the 1990s, from 6.5 attacks per year between 1990 and 1999 to 15 attacks per year between 2000 and 2009 [108]. Most of these attacks featured white (*Carcharodon carcharias*), tiger (*Galeocerdo cuvier*) and bull shark (*Carcharhinus leucas*). While in reality the risk of attack is very low, fear of sharks, especially the great white, has long been part of the Australian psyche [109]. The increase in attacks has led to controversial calls for protective measures, including, nets, baited drum lines and culling. A recent survey, carried out in Australia, highlighted that the public attitude towards sharks, following shark bite incidents, is generally positive, with a preference for nonlethal policies against shark attacks. Moreover, most of the respondents believed incidents to be accidental and not intentional [110].

Possibly related to their reputation as dangerous animals, sharks have often been been prized as trophies by professional and recreational fishermen, and their capture has been subsidized by governments [111]. For instance, between 1872 and 1909, the Austro-Hungarian Government established a reward for the capture of white sharks in the Kvarner Gulf (eastern Adriatic Sea) [112], where they were often seen or captured in the 'tonnare', traditional tuna traps used in the bluefin tuna fishery. The reward was proportional to the size of the captured white shark, and, in order to be paid, it was mandatory to bring the shark to the local maritime authority, open the belly and check for the presence of human bodies. Therefore, the capture of great white sharks was directly linked to the perceived dangerousness of the species.

The change in the perception of elasmobranchs also concerns the scientific world. Several elasmobranch species constitute the apex-predators in marine ecosystems. In the late 19^th^ and even early 20^th^ centuries, zoologists considered them, at least in some areas, to be voracious predators, consuming huge quantities of marine organisms and acting as competitors for fishers (see for instance [113]).

Only in recent times has the key role of elasmobranchs in shaping community structure and ecosystem functioning been scientifically recognized [1, 2, 5, 114–116]. Because many of these species are long-lived, and their biomass naturally fluctuates relatively slowly compared to other ecosystem components, these high trophic level predators help to stabilise ecosystems, principally by keeping the biomass of lower trophic levels in check [1]. This key role has been vividly demonstrated in comparisons of remote reef systems in the mid-Pacific, where the reef megafauna is intact and reef fish and coral diversity is high, with heavily-fished reef systems where large sharks are rare and fish and coral diversity is low [117, 118]. The important role of elasmobranchs, and in particular sharks, in maintaining healthy ecosystems is also starting to be conveyed to the public in documentaries (e.g. *Sharkwater* by Rob Stewart, 2006) and books (e.g. *If Sharks Disappeared* by Lily Williams, 2017). This role as ecosystem stabilisers has helped shift the public perception of megafauna in general and large sharks in particular from ‘brutish killers’ to necessary ecosystem actors [103].

#### Shift from extractive to non-extractive industries

Elasmobranchs, comprising sharks, skates, and rays, have a long history of exploitation, dating back at least to the Bronze Age [9, 119]. Even if they frequently represent the bycatch of more valuable species [120], specific fisheries targeting elasmobranchs were and are present in different areas, for the exploitation not only of their meat, but also of other body parts, such as the skin, or the liver to extract oil [121]. Moreover, their exploitation increased over time, also driven by the trade in highly valuable parts, such as fins, in several elasmobranch species, and gill plates, in manta rays [13, 122–124].

An emerging issue compromising shark conservation globally is the intentional mislabelling or erroneous labelling of shark meat [125]. Shark meat—in many cases of protected shark species—has been found labelled as flounder, tuna, or swordfish [125–127]. Both the U.S.A and the E.U. have rules for preventing fraud related to sharks and fisheries mislabelling. Nevertheless, there are serious concerns regarding the implementation of these measures. In addition, in many cases even if shark meat is labelled appropriately, the public may not be familiar with the common name used and may not be aware that they are buying shark meat.

In addition to commercial fishing, recreational fishing of sharks has been practised for decades. Even if this fishery is highly variable in space and time, shark catch by recreational fishermen may even exceed that of commercial fisheries in some areas, as for instance in the USA in 2013 [111].

Due to their exploitation, intentional or accidental mislabelling, and the vulnerability related to their life history traits, several populations of shark and skate are depleted, and, according to the IUCN Red List, one quarter of the species are threatened [13], mainly due to overexploitation [13, 128].

A change towards a non-extractive use of elasmobranchs has occurred in the last thirty years. In recreational fisheries, a change from killing to the practice of catch-and-release started in the 2000s in different parts of the world [111]. Moreover, even in commercial fisheries, modifications of fishing gears (net size, mesh design, hook size and type), to reduce the bycatch of sharks in fisheries targeting other other species, have been increasingly widely adopted worldwide [129]. However, data on shark survival rates both through the practice of catch-and-release and using methods to reduce the bycatch still improved are still insufficient [111, 129].

Their large size, elegant and powerful movements in the water, and, in some cases, even their ferocious appearance, have started to act as attractors, promoting a change in the use and value of these species [24, 76, 130]. Since the 1990s, shark tourism (we will use the terms 'shark watching' and 'shark tourism' to refer to all the activities related to the observation of sharks and their relatives, skates and rays, since this activity involves mainly sharks) has grown exponentially [131]. The shift in attitude towards sharks may be attributed to a number of factors, including diving magazine articles showing and describing their power and grace underwater, the development of appropriate viewing equipment such as shark cages, and shark feeding programs. Gradually sharks have become the holy grail of divers, attracting much of their attention. Today, the industry provides large economic returns for some local communities in coastal societies [24]. Gradually, articles, blogs, social media posts, videos and photos unravelled the “nice and fascinating” side of sharks; they are complemented by documentaries (e.g. *Sharkwater*, *Revolution*, *The Shark is still Working*“, *Extinction Soup*), aimed at educating the public and reversing (? abnegating / rethinking / countering) the 'beast' image of sharks. Some global scale reviews have highlighted the geographical coverage of shark watching, described the species involved, and quantified the economic value of this tourism activity [22, 24, 76, 131–133].

The attraction for sharks is not limited to shark watching in the field, but also involves large aquaria, which advertise shark tanks to attract visitors. Moreover, several shark-related activities, such as diving in or spending a night in front of the shark tanks, is a well-organized business activity at various aquaria in American, European and Asian countries.

Shark watching does not involve all elasmobranch species, but mainly species with certain characteristics. “As always, the bigger the better” [134] and “most of us became divers to see the big stuff” [135] are among the online phrases that best depict the main characteristics that a scuba diver searches for while looking for shark diving locations. To evaluate which species characteristics make a shark or a skate a preferential target for shark watching, we compiled a list of species reported in the literature as being a target for marine ecotourism. The size frequency distribution of focal species in shark watching was compared with the size frequency distribution of elasmobranchs in general (S2 Table). All data were extracted from Froese and Pauly [136], the most widely used and comprehensive database for fish studies. Moreover, for each target shark-watching species, we recorded: 1) habitat: coastal, oceanic, deep-water; 2) aggregative behaviour: occurring in small groups of just a few individuals, occurring in aggregations, or not aggregating; 3) IUCN status; 4) measures of protection; 5) whether it was responsible for unprovoked shark attacks: yes, no; 6) the perception of its dangerousness for humans: minimal, moderate, high-risk (as evaluated by [137]); and 7) geographical areas where watching occurs. To compile the information for each of these categories, we searched the scientific literature and databases [136, 138], and the international database of shark attacks (International Shark Attack File from the Florida Museum of Natural History, Museum of Florida, US).

**Fig 4:**
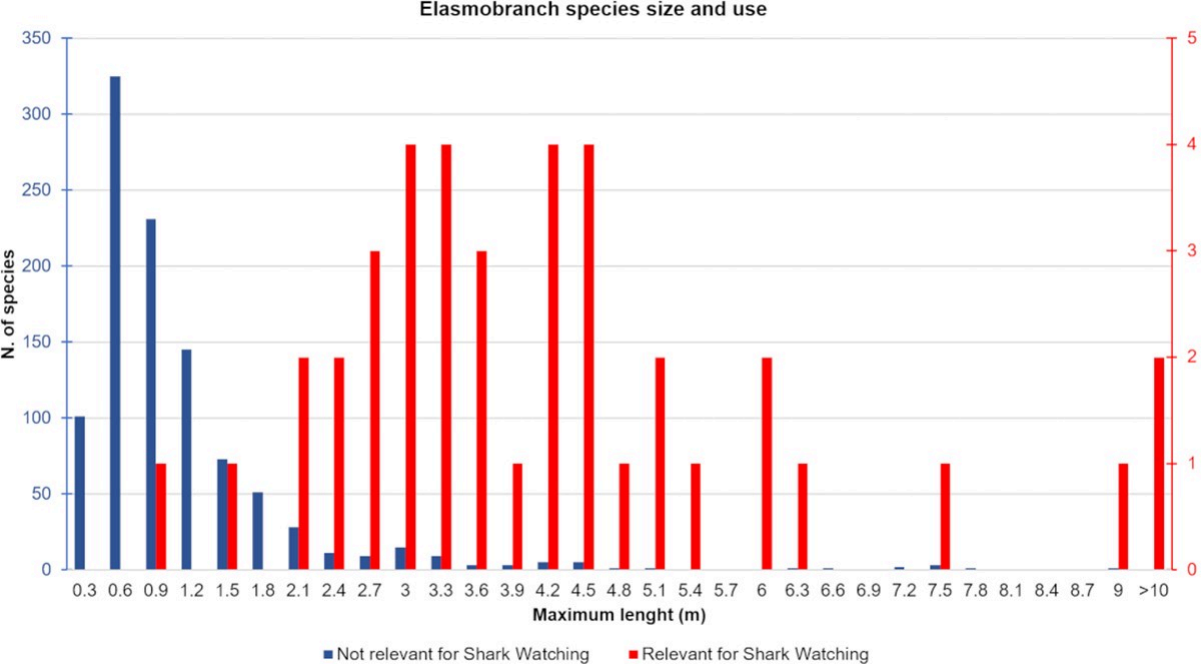
Size frequency distribution of elasmobranch species that are the target (in red) or not the target (in blue) of shark-watching activities (see S2 Table). Max length (m): maximum observed total length in m; N: number of species.

According to the available literature, at least 40 species, belonging to 16 families, are the target of shark watching activities (S3 Table). Most species (16) belong to the family Carcharhinidae. The size frequency distribution of species involved in shark watching is skewed towards large-sized animals (Fig 4), thus documenting people's interest in large animals, while small-sized ones are often neglected.

Not unexpectedly, most of the species (26) are reported to have a coastal distribution range, while eight are more oceanic. For five species, both habitats are reported, while one is reported to occur mainly in deep waters. The oceanic species include the thresher shark, the filter-feeding whale and basking shark, and the blue shark. While only a few sites are reported for watching thresher sharks [137], shark watching for the other species is mainly performed in areas where they predictably aggregate (filter-feeding species) or around seamounts (blue shark, see below).

**Fig 5:**
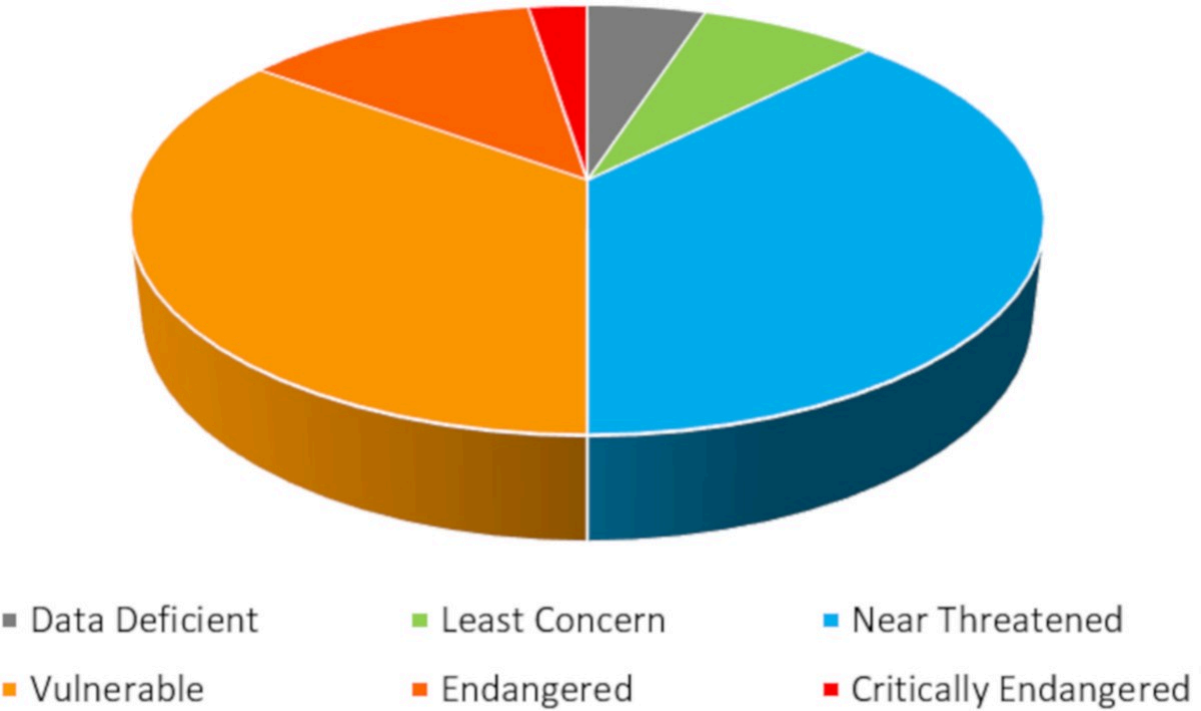
Percentage of species target of shark watching, that are included in the different categories of the IUCN Red List (see S3 Table for species data).

For 23 species, unprovoked shark attacks have been reported, and six species are considered of potential high risk to humans (S3 Table). With regard to their behaviour patterns, 24 species show aggregative behaviour (11 Carcharhinidae), five are reported to occur in small groups, while for 11 species no occurrence in groups or aggregations has been reported in the literature. Aggregations or groups may be associated with mating or feeding and may therefore be predictable in time and space [139]. This behaviour, together with site fidelity and residency in reefs, reported for some species [140–147], predispose them to be a target of shark watching. According to the IUCN, half of the species are threatened at some level, while 35% are near-threatened (Fig 5), and 16 species, representing 40% of species involved in shark watching, are under some form of protection (S3 Table).

Two contrasting examples may depict different routes, related to different perceptions, of this change in use: the white shark, on one hand, and the basking and whale shark, on the other.

The great white shark is probably the most popular and most iconic shark species worldwide, well known through several books, movies and documentaries, *Jaws* being the most popular among them. In this novel and the subsequent film, a white shark was portrayed as a ferocious animal and a man-eater. The novel and the movie helped to create a stereotype about white sharks, influencing a highly negative attitude towards sharks in general [148] and often triggering a shark cull culture among the fishing communities and the public [149]. Even documentaries, such as *Blue Water*, *White Depth* (1971), elicited a similarly negative attitude. Unfortunately, the negative image of the white shark created by *Jaws* became embedded in all the following movies. On the other hand, an increasing attraction for this terrifying species promoted a dedicated tourism business. White sharks were abundant in the waters off South Africa [150] where cage diving tourism developed shortly after the country enacted legislation to protect the species in 1991. This tourism activity increased over time, providing a significant economic contribution to the local community [131].

The whale shark (*Rhincodon typus*) and the basking shark (*Cetorhinus maximus*) are the largest species of shark and fish in general, reaching up to 20 m and 15 m in total length, respectively [136]. They are filter-feeding animals, preying on zooplankton and small fish while swimming with their large mouth open and filtering through the gill rakers, often close to the water surface. This feeding habit makes them harmless for humans. The basking shark is mainly distributed in cool temperate waters [136, 151, 152], while the whale shark occurs mainly in tropical and warm temperate areas, often along the coasts [136, 153, 154]. These two species have been exploited for centuries for their meat, but also for their liver oil, skin and fins [152, 154, 155]. Several characteristics make these species particularly attractive for shark watching. Their large size, occurrence close to the surface, and docile nature allow shark watching from boats, scuba diving and snorkelling [137, 156–158]. Moreover, both species tend to aggregate in specific places and seasons [139, 153, 154, 159, 160], making their encounter more predictable and therefore facilitating the organization of specific shark-watching activities. In several areas worldwide, an economically valuable ecotourism business dedicated to these species has been developed [137, 156–158] or is being developed [161].

#### Implications for conservation and management

A change in use from extractive to non-extractive, and specifically the development of tourism activities related to the watching of sharks and their relatives, may promote conservation, on one hand, and be limited by the lack of conservation actions, on the other. Here we provide some examples.

**Fig 6:**
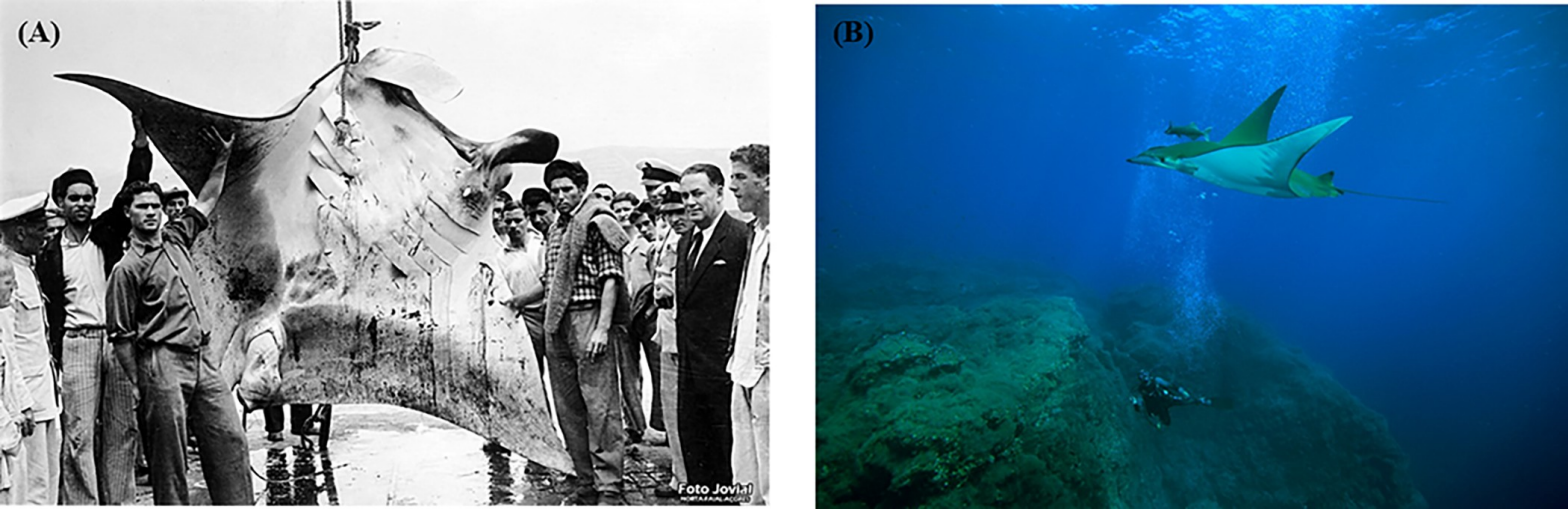
(A) Specimen of *Mobula birostris* harpooned in the 1950s, Horta harbour, (Faial island, Azores), reproduced under a CC BY license, by permission of the photographer, Foto Jovial-Horta-Faial-Açores; (B) Watching of mobula rays in the 2000s, Azores, reproduced under a CC BY license, by permission of the photographer, Jorge Fontes.

An example of how the tourism attraction of these animals may be a driver of conservation is represented by the manta and mobula rays. Manta and mobula rays include 11 species belonging to the genera *Manta* (2 species) and *Mobula* (9 species; [133]). These species may reach up to 7 m of disk width, inhabit different oceans and are filter-feeders [136]. Several species are known to aggregate for feeding, mating or visiting cleaning stations, often close to the water surface and in shallow waters [132, 133, 139, 159, 162, 163]. Manta rays have been heavily fished (Fig 6A) as bycatch or target species, in the last case mainly for their gill plates and branchial filaments, traded at high prices as medicinal products in some Asian communities [132, 133, 164]. In contrast, manta rays also constitute an attraction for snorkelers and divers (Fig 6B).

Their elegant swimming, their occurrence in shallow waters in predictable aggregations, and in addition their habit of leaping out of the water, make the observation of manta rays a target for divers. Nowadays, tourism related to manta watching has been developed in several areas, including the Central and Eastern Pacific Ocean, the Caribbean Sea, the Indian Ocean, the Western Pacific Ocean, and the Atlantic Ocean [133, 165]. Indonesia represented, up to 2014, one of the main countries fishing mantas for gill plates. In Indonesia, as well as other areas, the economic revenue related to manta watching has been increasing [132], leading in February 2014 to the establishment of a manta sanctuary in Indonesian waters. The important role of ecotourism pressure in promoting conservation may be represented by a comparison between manta and mobula rays. Much more effort has been devoted to the protection of mantas than mobulas, and this is probably related to the stronger development of tourism dedicated to manta watching than to mobula watching, and therefore to the relatively powerful charisma of the former compared to the latter. The stronger development of manta tourism has been partly due to the higher sighting frequency and reliability of manta rays, related to their more strikingly aggregative behaviour [166].

The development of the shark-watching business may also promote the establishment of marine protected areas (MPAs), as well represented by the example of the Azores archipelago and two shark species. The blue shark (*Prionace glauca*) and the shortfin mako shark (*Isurus oxyrinchus*) are highly migratory open-ocean predators, widely distributed across world’s oceans. These species are also an important fishery resource—either as a target or as a bycatch—for longline fisheries [167]. In the Azores archipelago, both shark species are an important bycatch for the pelagic longline fishery targeting swordfish [168, 169]. After the mid-1990s, having gained higher commercial value, they started to be landed [168]. Alongside their extractive use, these shark species have also a high non-extractive value for the regional marine ecotourism industry, attracting a significant number of divers. Diving with sharks for recreational purposes started recently, in 2011, mainly around the Faial and Pico islands, in the central group of the Azores [170]. It provides a challenging experience mainly accessible to experienced divers [170, 171]. Between 2011 and 2014, Condor seamount [172] represented the most important area in the region and one of the few in Europe for shark diving [170]. Like many seamounts elsewhere, Condor was a traditional fishing ground, especially for fishers from the Pico and Faial islands [173]. In 2010, Condor was designated as a scientific underwater observatory [174] and demersal fisheries were banned from the area. After the enforcement of the fishing ban, uses diversified towards lower-impact activities and recreational uses, and scientific research became progressively more important in the area. In fact, the fishing ban paved the way for the development of a new activity (i.e. shark diving) that had not previously existed in the region. At the same sites, recreational diving with mobula rays also started, around 2008 [170–172]. Mobula diving takes place mostly at offshore seamounts known to tourism operators and the fishing community as aggregation spots for mobulas [175]. In the Azorean archipelago, the presence of sharks and mobulid rays, allied to special management conditions (i.e. fishing ban) in force at Condor, acted as a catalyst for diversifying local livelihoods and provides a comprehensive example of a society’s change of perception and use regarding marine megafauna.

A clear example of the relationship between conservation status and the possible change in the use of shark species is represented by the Mediterranean Sea. In the Mediterranean Sea, a few sites are known and advertised for shark watching: Shark Point (Lebanon) for the sand tiger shark [137], Hadera (Israel) for the sandbar shark (*Carcharhinus plumbeus*) and the dusky shark (*Carcharhinus obscurus*) [176] and, more recently, Lampione Island (Pelagie Islands, Italy) for the sandbar shark, and the Strait of Messina for the sixgill shark, *Hexanchus griseus*. In the Mediterranean Sea, a strong decline in large-sized sharks, of more than 90% in a time range between 25 and 100 years depending on the species, has been documented [177, 178]. Moreover, medium-sized sharks have also declined, at least in some areas [179–181]. Today, the last report of the IUCN on the conservation status of sharks, skates and chimaeras in the Mediterranean Sea highlighted that more than half of the assessed species are at high risk of extinction [182]. This depletion or their local disappearance in specific places compared to the recent past (see [183] concerning the occurrence of sharks in the Aegean Sea), in particular of the large, more charismatic species, may impair the shift in use of these species in the Mediterranean Sea, i.e. there may be simply too few specimens left for reliable watching to be organised. In addition, some Mediterranean areas might be not suitable for promoting shark diving, such as the areas around Gaza where the occurrence of large aggregations of manta rays is well known [162], but tourism in general is still not fully developed due to the socio-political context.

As for cetaceans, there is increasing concern regarding the impact of unregulated shark-watching activities. Shark watching may induce the disruption of activities such as feeding, movements or social interaction, the alteration of behaviour patterns, physical injuries due to collision with boats, etc. [98, 184]. In addition, shark-watching activities may include provisioning, in the form of bait, a practice that may induce changes in the use of areas, aggressive behaviour towards conspecific individuals, and even a decline in body condition and animal health [95, 98].

### GROUPERS

#### Historical perspectives and human perceptions

The change in human perception and use has affected not only the most popular marine animals such as cetaceans and sharks, but also other species for which less literature is available, such as grouper.

Groupers (Family Epinephelidae, [185]) encompass a number of ecologically and economically important species worldwide. Most grouper are high-level predators inhabiting shallow coral or rocky reefs and continental shelves in tropical, subtropical and temperate waters [186]. Groupers are usually large-sized, long-lived and slow-growing species, well known for forming massive spawning aggregations at predictable times and places [186, 187].

Traditionally, groupers have been important solely as fishery resources. In the Mediterranean Sea, for instance, grouper have been caught since the end of the upper Palaeolithic era; bone remains indicate that they were well represented in the Neolithic, Greek and Roman times, and the Middle Ages [188, 189]. The maximum body size estimated from bone remains was approximately 90 cm, indicating that prehistoric humans could catch grouper of significant size [189]. In ancient times, as testified by Etruscan, Greek, and Roman paintings and mosaics, grouper might have been so large as to be portrayed as “sea monsters” [190]. In recent times, large grouper have been an attraction in public aquaria [191] as well as an appealing subject for documentaries and the earliest marine underwater explorations (see the film *The Silent World* [192], directed by Commandant J.Y. Cousteau). More recently, there has been increasing interest from divers. With the spread of recreational scuba diving and the near extinction of numerous species, growing interest has developed in admiring groupers as live attractions in natural surroundings. In fact, most of the biological traits that make grouper extremely vulnerable to fishing play also a crucial role in their rise in popularity among scuba divers. In particular, they are among the largest-sized reef fishes, site-attached with small home ranges, and form spawning aggregations that are predictable in time and space, thus maximizing dive viewing opportunities.

#### Shift from extractive to non-extractive industries

Grouper have been a fishery resource for centuries. Even in the present day, they are among the most commercially important and highly regarded fish species, representing a significant component of coastal fisheries at global scale [193].

The increase in sea-based activities sea-based recreational activities, primarily scuba diving, has driven a substantial change in the use of some grouper species, from extractive to non-extractive uses, with a significant increase in the overall generated income compared to that previously generated by fishing alone [194, 195]. In many countries, especially in tropical regions, this turnaround has already taken place [195, 196], while in others, such as in the temperate Mediterranean Sea, this trend is now getting under way [194]. In this respect, two case studies, one from tropical areas and one from the Mediterranean Sea, are presented here.

The Atlantic Goliath grouper, *Epinephelus itajara* (Lichtenstein, 1822), is the largest of the western North Atlantic groupers, reaching 400 kg and over 2 m in total length [197]. It is an amphi-Atlantic species, inhabiting a wide depth range, from shallow inshore waters to offshore depths of up to 100 m [197]. Natural as well as artificial reefs, rocky outcrops and shipwrecks are well-known aggregating sites for this species [198–201]. The Goliath grouper is of significant commercial and recreational interest [193, 197], and historical data from Florida Keys (i.e., photographs of ‘trophy fish’ and newspaper articles) provide evidence of population decline and nearshore depletion of the largest grouper since 1950 [202]. Since the late 1970s, the Goliath grouper has experienced a population collapse, becoming rare where formerly it was abundant [197, 203]; and overfishing has caused an extreme drop in abundance at aggregation sites throughout most of its range [197, 204]. Due to the above factors, this species is listed as Critically Endangered throughout its entire range in the IUCN Red List [205]. Its harvesting has been banned in south-eastern US federal waters since 1990, in the Caribbean since 1993 and in Brazil since 2002. Thanks to the fishing moratorium, since 1990, the number of Goliath grouper juveniles and adults has greatly increased in southern Florida, where extensive mangrove habitats have historically supported high densities of *E*. *itajara* and where a prehistoric fishery of the species existed [197, 206, 207]. Previously rare spawning aggregations of Goliath groupers became a source of revenue for the scuba diving sector [196]. In recent times, commercial boat operators in south-eastern Florida have expanded their businesses by taking divers out to see Goliath grouper aggregations [196, 198]. Socio-economic studies have found that a living Goliath grouper is more valuable than a dead one if one considers the revenues produced by diving tourism compared to fishing. Shideler et al. (2015) estimated that Florida recreational anglers’ perceptions and willingness to pay for a Goliath grouper harvest tag ranged between $34 and $79. Conversely, recreational divers are willing to pay around $100 for the first Goliath grouper sighting. This estimate increases to almost $170 and $336, for Florida and non-Florida divers, respectively, when dive trips encounter grouper aggregating for spawning [196]. Therefore, the economic value of Goliath grouper is significant especially during reproductive periods and this can potentially benefit local economies in the long-term, thanks to the long lifespan of this marine megafaunal species. This also implies that an eventual drop in Goliath grouper abundance and/or the disappearance of spawning aggregations would result in significant income loss in such communities [196].

Regarding temperate regions, the dusky grouper, *Epinephelus marginatus* (Lowe, 1834), is surely one of the most emblematic species in the Mediterranean basin. This species occurs in the eastern (including the Mediterranean Sea) as well as the southwestern Atlantic and western Indian Ocean [186, 193]. It mostly inhabits shelter-rich, hard substrates from the surface to 300 m [136], with maximum densities above 50 m depth [208].

The Mediterranean Sea has a long history of intensive exploitation [209] and the genus *Epinephelus* is no exception, being hunted for food for more than 10,000 years [189].

**Fig 7:**
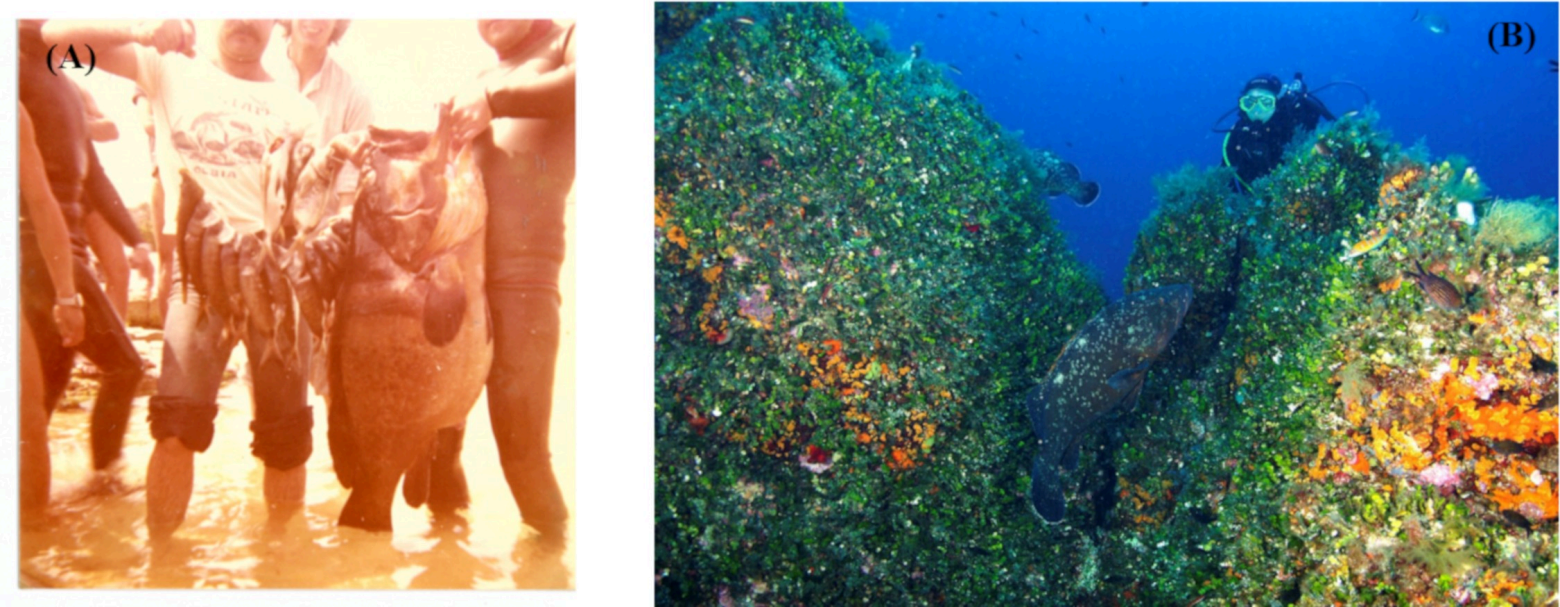
A) An old picture taken during a spearfishing competition held in 1977 in the area of Oristano (Sardinia, Italy, north-western Mediterranean Sea, reproduced under a CC BY license, by permission of the photographer Sergio Vitale; B) Scuba diver watching grouper in 2007 at the popular diving site Secche del Papa, in the Marine Protected Area of Tavolara-Punta Coda Cavallo (Sardinia, Italy, north-western Mediterranean Sea), reproduced under a CC BY license, by permission of the photographer Egidio Trainito.

Currently, *E*. *marginatus* is considered as overexploited in most Mediterranean areas and throughout its entire geographical range [193], since in recent decades it has undergone a severe drop in population size due to commercial as well as recreational fishing. It is particularly targeted by spear-fishing since its impressive bulk makes for a good trophy (Fig 7A). The historical increase in fishing effort has not only decimated this species but also caused a shift in the dusky grouper's depth range and size frequency of occurrence. In fact, in Mediterranean fished areas, large-bodied specimens are restricted to deeper waters while smaller individuals are more often encountered at shallow depths [190]. The dramatic decline undergone by this species lead to its being declared as 'Endangered' on the IUCN Red List for the Mediterranean Sea [210]. Some countries introduced fishing restrictions on the dusky grouper (e.g. since 1993, the ban on spear-fishing for dusky grouper along the French Mediterranean coasts, then extended to any recreational and professional fishing using hooks) and established coastal MPAs aimed at protecting marine communities, including threatened fishes such as the dusky grouper.

MPAs have proven to be effective in protecting and promoting the recovery of the dusky grouper [211, 212]. This positive outcome is mainly due to the life history traits of this territorial species [213], including strong site fidelity and small home range. This species is considered a reliable indicator of rich and diverse protected communities and it has been awarded with the title of 'Great Witness' since its abundance attests the success of the protection measures (*Grand Témoin*, [214]; Fig 7B).

#### Implications for conservation and management

Due to over-exploitation, many grouper populations have declined [215, 216]. Globally, 20 grouper species are currently classified as under threat of extinction in the IUCN Red List of Threatened Species [138]; and these estimates have remained unchanged since 2013 [216]. Catch-and-release recreational angling is promoted as a sustainable practice and conservation strategy for a diverse array of fishes, including grouper [217–220]. This fishing practice is sometimes permitted in protected areas, since anglers and fishery managers assume that the related mortality is negligible. However, this may not be the case, particularly for large-sized and long-lived species[218, 220] that may experience high, often unnoticed, levels of post-release mortality [219].

The change in use of groupers and the high value of live grouper have promoted the recovery of some populations, as highlighted by the case study of the Goliath grouper. However, despite the ongoing population recovery in eastern Florida and scuba diving businesses relying heavily on spawning aggregations, there is still cause for concern regarding this species. There is in fact no scientific evidence indicating that adult populations of Goliath grouper have fully recovered to pre-exploitation levels [221].

The recovery of megafauna populations may also elicit contrasting reactions in human communities. The return of adult Goliath groupers to certain reef locations and spawning aggregation sites off the south-eastern and south-western coasts of Florida [201, 221] has led to divergent and controversial use-oriented views and interests [222]. The observed population recovery has prompted a call for selective fishing of Goliath grouper, perceived as a nuisance by Florida fishers [197, 221, 223]. Some fishermen blame Goliath grouper for clearing reefs of prized crabs, lobsters and fish stocks, “anything they can vacuum up with their mammoth mouths” [224]. Commercial and recreational fishers have blamed Goliath grouper for gear damage and depredation, as well as for the decline of valuable fish prey and lobster stocks [223].

By comparison, the recovery of dusky grouper populations within effective Mediterranean MPAs has also contributed to the spread of the misconception that “there are too many grouper” (Desiderà, personal observation), to support their selective fishing. It is indeed a common belief among recreational fishermen that too many dusky grouper, as high-level predators, would cause the disappearance of prey such as octopus. Due to their position in the trophic web, grouper have been recognised as playing a key role in shaping community structure and ecosystem functioning [186]. Therefore, the protection and recovery of grouper populations may have significant ecological consequences through cascading effects [225]. In particular, the increase in high-level predator biomass and the restoration of predatory relationships could control the spread of some alien fish species [226]. In the Caribbean Sea, the density and biomass of the lionfish (*Pterois volitans* and *P*. *miles*) have been found to be lower in MPAs than in unprotected areas. Mumby et al. (2011, [227]) attributed these findings to the presence of more abundant and larger groupers (*Epinephelus striatus* and *Mycteroperca tigris*) inside MPAs, suggesting that groupers may act as a biocontrol of the invasive lionfish. Within Mediterranean MPAs, the role of groupers in controlling invasive species populations is not yet fully understood [228].

While recreational scuba diving might indeed be less destructive than extractive activities such as fishing, it does have a potential negative impact on wildlife and the environment. Dive tourism may include the intentional provisioning of fish to ensure close interaction with them [229, 230]. Concerns have been raised regarding the impact of this practice [230, 231]. Considering the dusky grouper, provisioning has been demonstrated to alter the species distribution [232]. Moreover, recreational fish feeding has been also found to promote an increase in dusky grouper density and an occasional diver-positive behaviour pattern, with just a few individuals approaching humans in the feeding locations [233]. Diving activities need to be regulated in order to reduce the risk that less well-trained and experienced divers might affect the behaviour of protected wild animals and/or impact fragile habitats.

## Discussion

In the past, cetaceans, elasmobranchs and grouper were considered predominantly resources for fishery/hunting exploitation. Moreover, in most westernized societies, many species were perceived as dangerous for humans and/or competitors for fishers, and therefore, in addition to being killed for exploitation, they were actively culled. At present, a growing interest in these species for ecotourism activities and as attractions in aquaria involves all the three groups. Elasmobranchs and grouper are still considered as valuable resources for fishers, with only a few species of elasmobranchs included in the appendices of CITES. However, a growing interest in protecting elasmobranchs has been expressed by the FAO International Plan of Action for Conservation and Management of Sharks, by international agreements (e.g. the ban on finning in European waters and by European fishing fleets; [234]), and by national and regional legislation in several countries. All cetacean species are included in appendix I or II of CITES (S1 Table), and whaling has been banned in most areas and countries of the world. However, subsistence whaling still occurs [235], and countries engaged in whaling, dolphin drive hunting, or other forms of cetacean hunting include Canada, the Faroe Islands, Greenland, Iceland, Indonesia, Japan, Norway, the Philippines, Russia, Saint Vincent and the Grenadines, the Solomon Islands, South Korea, and the United States.

**Fig 8:**
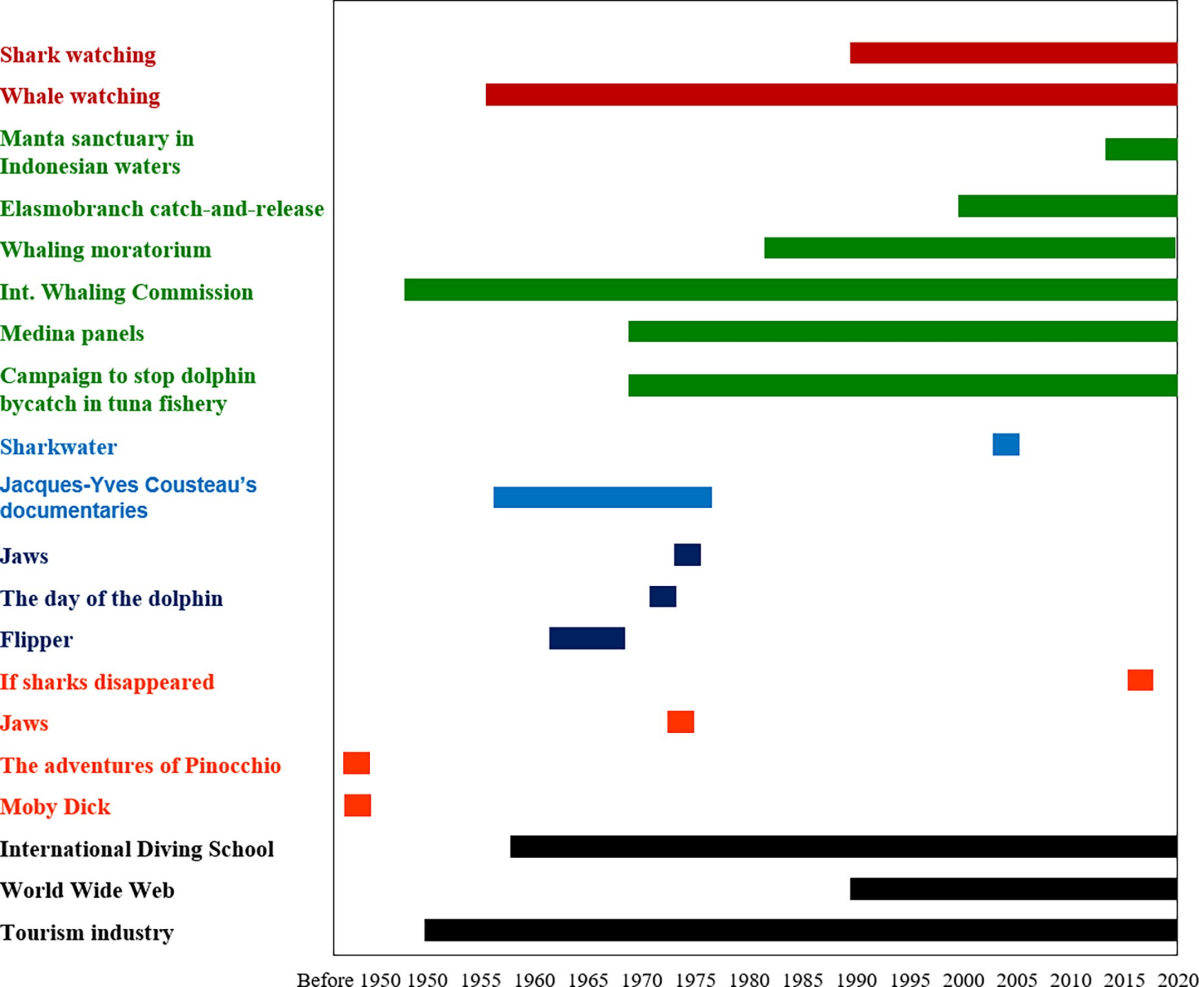
Graphical representation of the timescale of drivers and changes in perception and use of cetaceans, elasmobranchs and groupers. General drivers are presented in black, books in orange, movies and TV series in dark blue, documentaries in light blue, conservation action in green, whale and shark watching in red.

Common drivers for this change may be recognized (Fig 8). The screening of television documentaries on natural history starting from the 1950s (e.g. *Under the Sea* by Hans and Lotte Hass, *The Silent World* in 1956, and *The Undersea World of Jacques Cousteau* in 1966–1976), led to increased knowledge and appreciation of marine life. In the 1970s, environmental campaigns, aimed at raising public awareness with regard to certain charismatic species, such as small cetaceans, and pushing towards the reduction of their bycatch, began to develop. From the 1990s onwards, these campaigns were facilitated by the development and spread of new media, such as the World Wide Web. From the second half of the 20th Century, there was a boom in the scuba diving industry, with the foundation of international diving schools such as CMAS (World Confederation of Underwater Activities, founded in 1958), PADI (Professional Association of Diving Instructors, 1966), and SSI (Scuba Schools International, 1970). After the Second World War, the tourism industry developed and grew exponentially, due to the post-war economic boom and changes in lifestyle [236], opening the door to wildlife-watching activities. Nature-watching activities started by targeting cetaceans (since the 1970s), then sharks and grouper (from the 1990s). This time lag may be related to the fact that cetaceans are mainly observed from boats, while elasmobranchs and grouper normally require underwater watching; interest in these species grew along with the success of scuba diving.

The three groups of megafauna share some biological characteristics that might have influenced the change in use (Table 1). One of these shared characteristics is related to their large size. The attraction of large animals undoubtedly affects people’s choices (see also [237] for terrestrial animals). For example, large elasmobranch species are the main targets of shark watching. For species that are fishery targets, conversely, large size predicts vulnerability to exploitation [13, 238]. Consistently, large species showed the most marked decline in abundance [239]. Therefore, on one hand, the interest in large animals may support and promote their conservation, while on the other their abundance can be driven so low by exploitation as to impede the change towards non-extractive uses, as suggested by the low development of shark-watching in the Mediterranean Sea, where the conservation status of large-sized elasmobranchs is particularly poor [177].

**Table 1:** Comparison of the main characteristics of the three groups of megafauna.

	Cetaceans	Elasmobranchs	Groupers
Change in perception	Yes (whales), Partial (dolphins, still perceived as competitors)	Partial (fear still occurring)	Partial (still considered as trophy fish and/or predator to be culled)
**Beginning of change in perception and use**	1970’	1990’	1990’
**Beginning of conservation measures**	1975	2001	Early 1990’
**Main type of watching**	From boat	Underwater	Underwater
**Biological characteristics: large size**	Most species are large, not relevant	Relevant	Relevant
**Biological characteristics: site fidelity**	Relevant	Relevant	Relevant
**Biological characteristics: occurrence in groups or aggregations**	Relevant	Relevant	Relevant
**Change in value (non-extractive use vs extractive)**	Higher value	Higher value	Higher value
**Negative impact of ecotourism**	Documented	Documented	Documented

Successful sea-life watching requires encounters with target species that are predictable in time and space. Site fidelity is particularly highly developed in grouper, that use specific areas for feeding, spawning, giving birth or as a nursery, and aggregative and/or social behaviour patterns, occurring in all the three groups, fulfil these requirements (Table 1).

In all case studies, the different economic value of a dead (extractive use, fishery) or an alive (non-extractive use, ecotourism) specimen, has been widely documented (e.g. [24, 26, 59, 196, 223]). In some cases, it has been claimed that a single live cetacean, elasmobranch, or grouper may have a much higher value than a dead one [26, 240]. More generally, the tourism value of wildlife can undoubtedly bring more economic benefit than extractive uses, and therefore has the potential for promoting species conservation [241].

The change from extractive to non-extractive use should promote species conservation through different mechanisms [131, 184, 242]. A direct mechanism is related to the revenue from the new non-extractive use, that may push towards the establishment of conservation action [131], as documented by the Manta ray case study, or the Bahamas elasmobranch case studies [25]. A link between conservation and the change in use is also documented by the tourism attraction exerted by MPAs [243, 244]. A second mechanism concerns the educational value of ecotourism activities, as wildlife watching may positively influence tourist attitudes [184, 242]. However, a fundamental additional aspect relates to the beneficiaries of the change in use of marine megafauna. The change from extractive to non-extractive use may or may not involve the same stakeholders and communities. In some areas, fishers resorted to ecotourism, with direct revenues from this change in use. In Western Australia, for instance, charter fishing and ecotourism frequently developed as seasonal side occupations for professional fishers. In the 1990s, about half of all charter boat operators simultaneously held commercial fishing licenses [245]. In other cases, tourists pay compensation to local communities of fishers for the loss of fishery opportunities resulting from conservation-oriented management [242, 246]. In most cases the transition from extractive to non-extractive uses of marine megafauna does produce greater benefits, but these benefits cascade down to different stakeholders. So here the point is not just the fact that individual stakeholders may have conflicting needs and interests [247]. The point is that benefits consequent to such a transition may be funnelled from some stakeholders (sometimes socio-economically more vulnerable, such as local fishers) to others (often more organised from an entrepreneurial point of view, e.g. tour operators, diving centres), including 'external' stakeholders, moving in from other areas to exploit new opportunities. 'Free access' to the resources for ecotourism may limit the opportunities for local communities and have implications also for the sustainability of these activities from a conservation point of view [248]. Changes in use, if not accompanied by appropriate compensation measures ('transition assistance'), that in the short to medium term aim to assist with the adjustment or re-orientation of activities [249], may trigger strong opposition from the ‘economic losers’ and reduce the local level of compliance towards conservation/management measures regarding megafauna. In a broad meta-analysis on the role of ecotourism in conservation [19], Krüger indeed highlighted a close link between the success of conservation and the involvement of local communities in the ecotourism activities.

Ecotourism activities cannot be considered an easy solution for conservation. Indeed, they may have a negative impact on the targeted species. Wildlife watching, for example, either from boats or underwater, may have detrimental effects on marine wildlife [95, 98]. The traffic and noise of whale- and dolphin-watching boats may interfere with animal behaviour, communication and energy expenditure [97, 250, 251]. Provisioning of food to attract animals may affect behaviour, diet, animal health and condition and reproductive success [95, 98, 184, 252–257]. As a consequence of the increasing evidence of the impact of tourism activities on wildlife, ethical concerns have been raised regarding these activities [94], and codes of conduct are being developed for the different activities, areas, and species involved [75, 258, 259]. Moreover, it has been documented that uncontrolled ecotourism activity may indeed impair the expected positive consequences of the reduction in extractive uses [19].

Despite these conservation concerns (that appear relatively minor when compared with hunting and overexploitation), the change in the perception of marine megafauna over just a few human generations—from monsters to be feared and exterminated, to beautiful creatures to be observed and admired—is striking. This change leaves room for hope that humankind will continue to develop its more positive attitude towards wildlife and the ecosystems that sustain humans on planet earth.

As outlined in the previous sections, the change of use, from exploitation to observation, outlined for the three groups, is not universal and does not involve all the species or geographical areas in the same way. Moreover, all the three groups are impacted not only by intentional or unintentional capture, but also by other drivers impacting marine species and ecosystems in general (including pollution, habitat degradation and climate change) [260]), Clearly, ecotourism does not represent a complete 'solution' for megafauna conservation [19]. However, we believe that it can represent a powerful driver contributing to the adoption of conservation measures mitigating at least some of the impact affecting marine megafauna.

## Supporting information

S1 Table Cetacean species target of whale watching worldwide, as reported in [1]. The conservation status, according to the Red List of the IUCN, and the protection measures, according to CITES, have been included.
(XLSX)

S2 Table Maximum size of elasmobranch species. TL: total length. All data from: Froese R, Pauly D. Editors. FishBase. World Wide Web electronic publication. www.fishbase.org, version (02/2018); 2018.
(XLSX)

S3 Table Characteristics of elasmobranch species target of shark watching.TL: Total length. (XLSX)
